# Carob (Ceratonia siliqua L.) fruit hydro-alcoholic extract alleviates reproductive toxicity of lead in male mice: Evidence on sperm parameters, sex hormones, oxidative stress biomarkers and expression of *Nrf2* and *iNOS*

**Published:** 2020

**Authors:** Ali Soleimanzadeh, Mehdi Kian, Sajjad Moradi, Soraya Mahmoudi

**Affiliations:** 1 *Department of Theriogenology, Faculty of Veterinary Medicine, Urmia University, Urmia, Iran*; 2 *Department of Pathobiology, Faculty of Veterinary medicine, Urmia University, Urmia, Iran*

**Keywords:** Ceratonia siliqua, Pb poisoning, Oxidative stress, Lipid peroxidation, Nrf2, Inducible nitric oxide synthase, Male reproductive system

## Abstract

**Objective:**

Carob *(Ceratonia siliqua L.)* is an evergreen tree with fruits that have potent antioxidant activity. The aim of this study was to investigate alleviative effects of carob fruit hydro-alcoholic extract (CFHAE) against reproductive toxicity induced by lead (Pb) in male mice.

**Materials and Methods:**

Forty-two NMRI adult male mice were randomly categorized into 7 groups (N=6). Group I was the control group and received no treatment. Group II was the sham group and received 0.2 ml distilled water per day. Group III (Pb group) received Pb acetate 1000 ppm/kg/day. Groups IV and V received CFHAE 500 and 1000 mg/kg/day, respectively. Groups VI and VII received both Pb 1000 ppm/kg/day and CFHAE at doses of 500 and 1000 mg/kg/day, respectively at the same time. The groups were treated by gavage. After 35 days, sperm parameters (count, motility, morphology, viability, DNA damage, and teratozoospermia index), total antioxidant capacity (TAC), reduced glutathione content (GSH), antioxidant enzymes (SOD, CAT, and GPx) activity, MDA levels, and sex hormones (FSH, LH, and testosterone) concentrations in serum, testicular expression of *Nrf2* and *iNOS* genes and histopathological alterations were evaluated.

**Results:**

Our findings revealed that co-administration of CFHAE with Pb significantly (p<0.05 to p<0.01) improved sperm parameters, elevated sex hormones, TAC, GSH content, and antioxidant enzymes activity of serum, decreased serum MDA levels, and down-regulated testicular expression of *Nrf2* and *iNOS* genes compared with Pb group. Also, CFHAE ameliorated histopathological alterations in testis tissue caused by Pb.

**Conclusion:**

CFHAE can alleviate reproductive toxicity following Pb exposure in male mice.

## Introduction

Infertility is considered one of the most important issues in married couples. It has been estimated, a male factor infertility plays a role in about 50% of all infertility cases (Sigman, 2007 ▶). Environmental exposures to toxicants are directly associated with male infertility (Wong and Cheng, 2011 ▶). Lead (Pb) is an abundant toxic heavy metal in the environment (Bae et al., 2001 ▶; Shotyk and Le Roux, 2005 ▶). This heavy metal is widely used in industries and present in many products and some cosmetics (Ayinde et al., 2012 ▶; Chowdhury, 2009 ▶). Human exposure to Pb could occur via air, water, soil, food, and consumer products (Hammond, 1977 ▶).

Evidence elucidated that Pb is toxic for male reproductive system. Exposure to Pb causes generation of reactive oxygen species (ROS), lipid peroxidation and depletion of antioxidants which lead to induction of oxidative stress in testis tissue (Moniem et al., 2010 ▶; Reshma Anjum et al., 2011 ▶; Ayinde et al., 2012 ▶; Reshma Anjum et al., 2017 ▶; Elgawish and Abdelrazek, 2014 ▶). Also, Pb attenuates sperm parameters and diminishes the activity of spermatozoa (Chowdhury, 2009 ▶). Pb exposure impairs sperm normal function by decreasing the levels of sperm intracellular cyclic adenosine monophosphate and calcium and reducing tyrosine phosphorylation of sperm proteins (He et al., 2016 ▶). Many studies indicated that administration of antioxidant herbal plants can alleviate oxidative stress induced by Pb in experimental models (El-Nekeety et al., 2009 ▶; Dorostghoal et al., 2014 ▶; Dkhil et al., 2016 ▶; Soleimanzadeh et al., 2018 ▶). 

Carob (*Ceratonia siliqua L.*) which belongs to the *Fabaceae* family is an evergreen tree cultivated in the Mediterranean countries (Dakia et al., 2007 ▶; Özcan et al., 2007 ▶). This plant grows widely in Fars province, Iran (Mokhtari et al., 2012 ▶; Vafaei et al., 2018 ▶). The carob fruit is a brown pod with length of 10–25 cm that consists of 80–90% pod and 10–20% seed by weight (Naghmouchi et al., 2009 ▶; Tetik et al., 2011 ▶). Carob fruit possesses high amounts of antioxidant compounds such as flavonoids and polyphenols and has strong radical scavenging activity (Custódio et al., 2009 ▶; Kumazawa et al., 2002 ▶; Owen et al., 2003 ▶; Papagiannopoulos et al., 2004 ▶). Previous studies indicated protective effects of carob against oxidative stress in different organs (Abdel-Rahman et al., 2018 ▶; Rahman et al., 2016 ▶; Rtibi et al., 2016a ▶, 2016b, 2015; Suzek et al., 2017 ▶; Vafaei et al., 2018 ▶).

In Persian traditional medicine, carob fruit is used as an aphrodisiac and to increase human semen volume to treat male infertility. Recently, it has been demonstrated that aqueous extract of carob pod has a beneficial effect on male reproductive parameters in infertile mice (Vafaei et al., 2018 ▶). In another study done by Mokhtari et al., administration of carob seeds hydro-alcoholic extract increased levels of sex hormones and sperm density in seminiferous tubules (Mokhtari et al., 2012 ▶). Also, it has been reported that carob pods extract has a possible positive influence against Pb toxicity and may interfere with the absorption of Pb (Said et al., 2017 ▶). Hence, in the present study, possible alleviative effects of carob fruit hydro-alcoholic extract (CFHAE) on Pb -induced oxidative damages on male reproductive parameters, were evaluated.

## Materials and Methods

**Animals**

Forty-two, 8-10-week-old NMRI male mice (30±6 g) were purchased from the animal house of the Urmia Medical University, Iran. The animals were maintained under standard laboratory conditions (with 12:12 hr light/dark cycle at 25±2°C). They were acclimatized for one week before the study and were provided with tap water and standard laboratory diet *ad libitum*. The experimental protocols were approved by the Animals Ethics Committee at Urmia University (AECVU-170-2018).

**Plant extract preparation**

Carob fruits were purchased from a Persian herbal market and their identity was confirmed in Natural Resource Center (Herbarium No. 7123). The fruits (seeds+pods) were ground to a fine powder and soaked in 96% ethanol (Merck, Darmstadt, Germany) and water (50:50 ratio) for 72 hr. Whatman filter No. 40 was utilized to filter the extract; after that, the extract was concentrated at 50°C using a rotary evaporator (Heidolph Laborota 4000 efficient, Schwabach, Germany). The remaining brown pasty extract was dried in an oven at 40°C (Mokhtari et al., 2012 ▶). Eventually, the CFHEA was maintained at -20°C in order to use in subsequent *in-vitro* and *in-vivo* experiments.

**HPLC analysis to identify and quantify phenolic and flavonoids compounds**

Based on a modified version of Singleton’s method by Dewanto et al. (Dewanto et al., 2002 ▶), the Folin-Ciocalteu reagent was used to analyze the phenolic contents. One aliquot (0.125 ml) of a desirable methanolic extract which was diluted, was poured into 0.5 ml of deionized water which contained 0.125 ml of Folin-Ciocalteu reagent. Then, 1.25 ml of 7% Na_2_CO_3_ was added to the mixture, and the solution was shaken and left for 6 min before adding Na_2_CO_3_. In order to increase the final volume of the solution to 3 ml, deionized water was added. Afterwards, the mixture was totally stirred. The absorbance was read at 760 nm versus prepared blank following incubation for 90 min at 23°C. 

Agilent Technologies 1100 series liquid chromatograph (Reversed phase HPLC) coupled with an UV–vis multi-wavelength detector, was adopted to analyze the phenolic compound. With a 250×4.6 mm, 4 µm Hypersil ODS C18 reversed-phase column at ambient temperature, the separation was performed. The injected volume was 20 µl and prior to injection, the samples were filtered through a 0.45 µm membrane filter and peaks were monitored at 280 nm. The phenolic compounds were distinguished from other components based on their retention duration, spectral features of the peaks versus the standards and transfixing the sample with standards. The Analyses were carried out with three replications.

**Experimental design**

The animals were categorized into 7 groups and each group included 6 animals. Group I was the control group and received no treatment. Group II was the sham group and received distilled water per day by gavage. Group III (Pb group) received Pb acetate (Sigma-Aldrich, USA; CAS Number 6080-56-4) 1000 ppm/kg/day by gavage. Groups IV and V received CFHAE, 500 and 1000 mg/kg/day, respectively by gavage. Groups VI and VII received Pb 1000 ppm/kg/day and CFHAE at doses of 500 and 1000 mg/kg/day, respectively by gavage at the same time. The animals received treatments for 35 consecutive days. The dose of Pb was chosen based on Soleimanzadeh et al. (2018) ▶. 

**Plasma sampling**

After 35 days, animals were anesthetized using intraperitoneal administration of a combination of 2% xylazine (10 mg/kg, Alfasan, Woerden, Holland) and 5% ketamine hydrochloride (90 mg/kg, Rotexmedica, Trittau, Germany) according to Kanter (2010) method. Then, animals were sacrificed by cutting their necks, blood samples were collected in laboratory tubes and centrifuged at 3000 g for 15 min. The separated serum samples were stored in microtubes at -20°C until used for biochemical and hormonal evaluations (Soleimanzadeh et al., 2018 ▶).

**Collection of epididymal sperms**

Sperm samples were obtained from the cauda epididymis of the testes of each mice. The cauda epididymis was excised into small pieces and placed in Petri dishes containing 1 ml of Human Tubal Fluid (HTF) medium at 37°C for 30 min.

**Sperm motility**

Assessment of the sperm motility was performed according to the WHO laboratory manual protocol for the examination of human semen (2010). In brief, 10 μl of the sperm suspension was placed on a semen analysis chamber. A minimum of five microscopic fields were assessed in terms of sperm motility on at least 200 sperm for each animal.

**Sperm viability**

Eosin-Nigrosine staining was used to assess sperm viability according to the WHO protocol (World Health Organization, 2010 ▶). Briefly, Eosin (Merck, Darmstadt, Germany) and Nigrosine (Merck, Darmstadt, Germany) were prepared in distilled water. One volume of sperm suspension was mixed with two volumes of 1% eosin. After 30 sec, an equal volume of nigrosine was added to this mixture. Thin smears were then prepared and observed under a light microscope (Model CHT, Olympus optical Co. Ltd., Tokyo, Japan) at 1000X magnification. Viable sperm remained colorless while nonviable sperm was stained red.

**Teratozoospermia** **i** **ndex (TZI)**

The TZI or multiple anomalies index is the number of defects per abnormal spermatozoon. Each abnormal spermatozoon may have one to four abnormalities including head, neck/mid piece and tail defects or presence of cytoplasmic residues. TZI values have been read between 1.00 (each abnormal spermatozoon has only one defect) and 3.00 (each abnormal spermatozoon has a head, midpiece, and tail defects) as described by Krassas et al. (Krassas et al., 2008 ▶).

**Assessment of DNA damage**

Sperm suspensions were washed 3 times with 5 ml of phosphate buffered solution (PBS) and centrifuged at 3000 rpm for 5 min. Thin smears were prepared from the sperm solution and allowed to air-dry. To test sperm DNA integrity, the smears were stained with Acridine-Orange (AO). The AO staining was performed according to a protocol described by Tejada et al. (Tejada et al., 1984 ▶). In brief, the smears were fixed for 24 hr in methanol/acetic acid (3:1) at 4°C and stained with AO solution (0.19% in phosphate-citrate buffer, pH 2.5) for 10 min. The slides were gently washed by distilled water for 5 min and air dried. The stained smears were then observed under a fluorescence microscope (Model GS7, Nikon Co., Tokyo, Japan) at 1000X magnification. Three types of staining patterns were considered in sperm head; green spermatozoa (double-stranded DNA), and yellow and red spermatozoa (single-stranded DNA). At least 100 spermatozoa per slide were counted to evaluate the percentage of double-stranded DNA in the spermatozoa (Tejada et al., 1984 ▶).

**Estimation of total antioxidant capacity (TAC)**

The TAC of the serum was estimated by ferric reduction antioxidant power (FRAP) assay (Benzie and Strain, 1999 ▶). Here, 100 µl of cellular supernatant was added to 1 ml of fresh ferric reducing antioxidant power reagent (FRAP; Tripiridyltriazine; Merck) and incubated at 37°C for 10 min in the dark. Reading of the blue-colored reagent was done at 595 nm every 20 sec for 10 min. An aqueous solution of FeII (FeSO_4_.7H_2_O) and appropriate concentrations of freshly prepared ascorbic acid were used as blank and standard solutions, respectively. The results were expressed as μmol of TAC per L serum.

**Estimation of** **reduced glutathione (GSH)**

Glutathione (GSH) was assayed using a modified method described by Beutler et al. (1963) ▶. In brief, 0.9 ml distilled water and 1.5 ml of precipitating reagent were added (3.34 g metaphosphoric acid, 0.4 g EDTA, and 60.0 g sodium chloride) to 0.1 ml of sample. The tubes were vortexed and left for 5 min at room temperature. The solution was centrifuged for 15 min at 4000 rpm at 4°C. Next, 4.0 ml of phosphate solution (0.3 M disodium hydrogen phosphate) and 0.5 ml 5-50-dithiobis-(2-nitrobenzoic acid) (DTNB) (80 mg in 1% sodium citrate) were mixed with 1.0 ml supernatant. The absorbance of the mixture containing a yellow color complex was read immediately at 412 nm spectrophotometrically. A standard curve for GSH was prepared and GSH concentration in the experimental samples was extrapolated from the standard curve. The results were expressed as U/L serum. 

**Measurement of** **enzymatic** **antioxidan** **ts** **activity**

The activity of superoxide dismutase (SOD) was measured in terms of inhibition of the nicotinamide adenine dinucleotide (reduced)-phenazinemethosulphate-nitrobluetetrazolium reaction system according to the Nishikimi et al. (1972) ▶ method. The catalase (CAT) activity was determined based on the reduction of dichromate in acetic acid to chromic acetate when heated in the presence of H_2_O_2_ according to Sinha (1972) ▶. The activity of glutathione peroxidase (GPx) was estimated according to the method described by Paglia and Valentine (1967) ▶ based on the fact that GPx catalyzed the oxidation of glutathione by cumene hydroperoxide. The results were presented as U/L serum. 

**Lipids peroxidation measurement**

The rate of lipid peroxidation in the serum samples was estimated by determination of malondialdehyde (MDA) using the thiobarbituric acid reactive substances (TBARS) test (Placer et al., 1966 ▶). The results were presented as µmol/L.

**Hormonal assessments**

Serum concentration of testosterone was measured by enzyme-linked immunosorbent assay (ELISA) as described in the instructions of the manufacturer’s kit (Demeditec Diagnostics GmbH, Germany). The results were presented as μmol/L serum.

Serum levels of LH and FSH were determined by ELISA using specific commercial kits (Amersham, Buckinghamshire, UK) according to Loraine and Bell, 1971 methods. The results were presented as mIU/ml serum.

**Histopathological evaluations**

Right testes of animals were fixed in formalin 10% solution. Then, the samples were dehydrated by a graded series of ethanol and embedded in paraffin. Thin sections were cut by microtome into 7 μm thickness. Next, prepared sectioned were stained by hematoxylin and eosin (H&E) staining according to the method described by Suvarna et al. (2018) ▶. The stained sections were evaluated for histopathological alterations by using a light microscope (Olympus Model BH-2, Tokyo, Japan). 

**RNA extraction**

Using Cinna Pure-RNA kit (CinnaGen, Cat. No. PR891620, Iran), the total RNA of the left testis of mice was extracted. ND-2000 spectrophotometer (Nanodrop Technologies, Thermo Fisher Scientific, USA) was used to determine concentration and purity of the total RNA and then the total RNA was stored at -70°C until cDNA synthesis. 

**cDNA synthesis**

**Figure 1:**
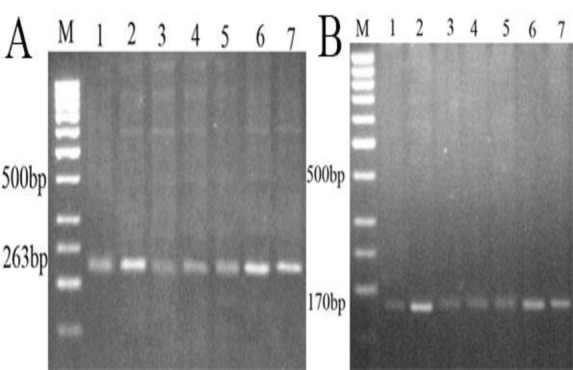
Primer specificity and cDNA synthesis products. A) Nrf2 (263 bp), B) and iNOS (170 bp) gene primer; Lane M: 100 bp DNA ladder, Lane 1, Control group; Lane 2, Pb group; Lane 3, Sham group; Lane 4, CFHAE 500 group; Lane 5, CFHAE 1000 group; Lane 6, Pb+CFHAE 500 group; Lane 7, Pb+CFHAE 1000 group

Using random hexamers primers (Primer+dNTP Mix+Nuclease-free water), first strand cDNA was synthesized from 10 μg of total RNA and cDNA Synthesis Mix (M-MLV Reverse Transcriptase+10X Buffer M-MuLV+Nuclease-free Water) (Vivantis Technologies, Cat. No. RTPL12 100 app, Malaysia) was incubated at 42°C for 60 min and the reaction was terminated by incubating at 85°C for 5 min followed by cooling at 4°C. Synthesized cDNA was stored at -20°C until processed. The electrophoresis was performed with the PCR products to verify the primers specificity (Figure 1). 

**Quantitative** **r** **eal-** **t** **ime PCR assay**

Using SinaSyber Blue HF- qPCR Mix (CinnaGen, Cat. No. MM2171, Iran) on a StepOne™ real-time PCR system (Applied Biosystems, USA) using quantitative real-time polymerase chain reaction (qRT-PCR), the mRNA expression levels of *Nrf2*, *iNOS* and *18SrRNA* were quantified. The characteristics of the primer pairs used in the present study, are presented in Table 1. Reactions included 12.5 μl of SinaSyber Blue HF- qPCR Mix (CinnaGen, Cat. No. MM2171, Iran), 0.25 μl of each primer (2 μM), 2 μl of cDNA, 9.5 μl of nuclease-free water at 95°C for 10 s, followed by 40 cycles of 95°C for 10 s, and 60°C for 30 s.

A standard curve from the cDNA reaction mixture was used to detect PCR efficiencies. The efficiencies of PCR were between 90 and 110 percent. The amounts for R2 for all curves were observed from 0.997 to 0.999. The amounts of CT and expression of the relative expression level of the target gene as 2-CT were used for quantification of the product of PCR. Following, the CT was measured ΔCT after subtracting CT of the housekeeping gene (*18SrRNA*), from CT of the target gene.

**Statistical analysis**

Statistical analysis was performed using SPSS version 21 (IBM Co., Chicago, USA). The data were analyzed by one-way ANOVA and the Tukey test was used as *post hoc*. A p value of less than 0.05 was considered significant.

## Results

**Phytochemical** **analysis**

The phytochemical investigation using RP–HPLC method revealed various phenolic and flavonoid compounds in CFHAE, as shown in Table 2. The compounds are including gallic acid, caffeic acid, chlorogenic acid, rutin, coumaric acid quercetin, cinnamic acid, and apigenin.

**Table 1:** The characteristics of primer pairs used in the present study

**Name/gene**	**Primer sequence**	**Ref. sequence (accession number acc. to GeneBank)**	**Product length (base pairs, bp)**
***iNOS***	F: 5′ CACCTTGGAGTTCACCCAGT 3′ R: 5′ ACCACTCGTACTTGGGATGC 3′	NM_010927	170 bp
***Nrf2***	F: 5′ CACCTTGGAGTTCACCCAGT 3′ R: 5′ ACCACTCGTACTTGGGATGC 3′	NM172086	263 bp
***18SrRNA***	F: 5΄GCAATTATTCCCCATGAACG 3′ R: 5′ GGCCTCACTAAACCATCCAA 3′	NR_003278	123 bp

iNOS: Inducible Nitric Oxide Synthase; Nrf2: nuclear factor erythroid 2–related factor 2

**Effects on s** **perm parameters**

**Figure 2:**
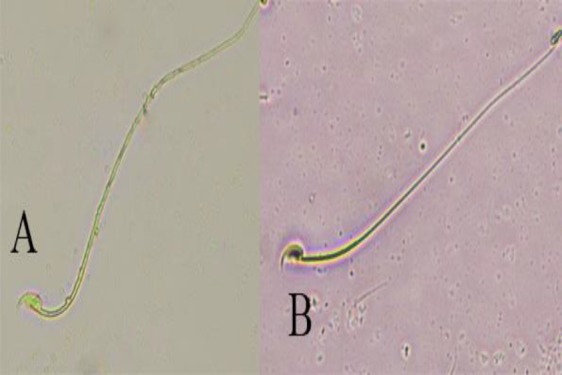
Sperm viability in groups; A) Viable sperm (colorless), B) Dead sperm (red); (eosin/nigrosine, 1000X)

**Figure 3:**
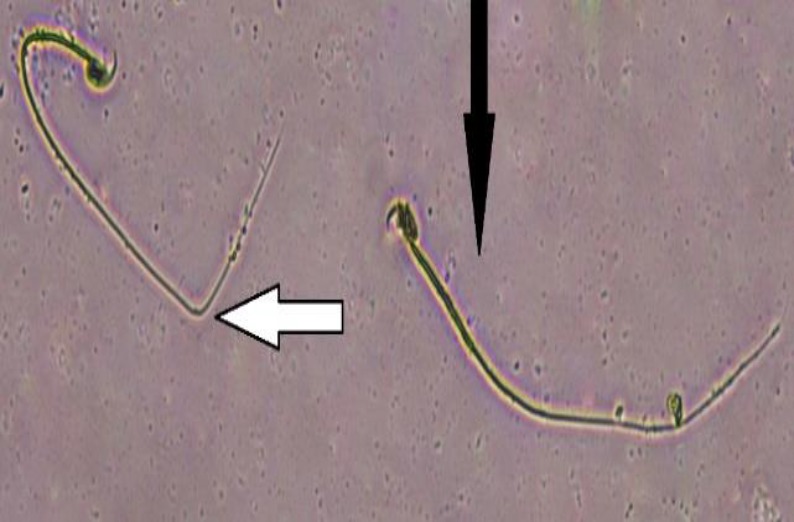
Morphologically normal and abnormal sperms in groups; normal (black arrows), and abnormal (white arrows); (eosin/nigrosine, 1000X)

**Figure 4:**
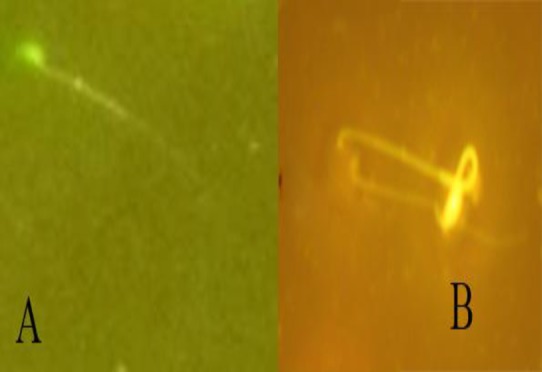
DNA damage in groups; A) Normal sperm (green), B) Damaged DNA (yellow); (AO, 400X)

Data summarized in Table 3 show that exposure to Pb significantly (p<0.01 for all cases) attenuated sperm parameters compared to the control group but co-administration of CFHAE and Pb significantly (p<0.05 to p<0.01) enhanced these parameters compared with Pb group (Figures 2-4). 

**Effects on TAC, GSH content and antioxidant enzymes activity in serum**

Exposure to Pb significantly (p<0.01 for all cases) decreased TAC, GSH content and activities of enzymatic antioxidants (SOD, CAT, and GPx) in serum compared to control and sham groups while co-administration of CFHAE and Pb significantly (p<0.05 to p<0.01) and dose-dependently increased them in comparison with Pb group (Table 4).

**Table 2:** The amounts of total phenolic and flavonoid compounds in CFHAE as determined by HPLC analysis

**Compounds**	**mg/kg**
Gallic acid	408.37
Caffeic acid	100.58
Chlorogenic acid	123.58
Rutin	89.25
Coumaric acid	121.81
Quercetin	88.68
Cinnamic acid	20.80
Apigenin	62.38

**Effects on serum** **lipid peroxidation**

**Figure 5:**
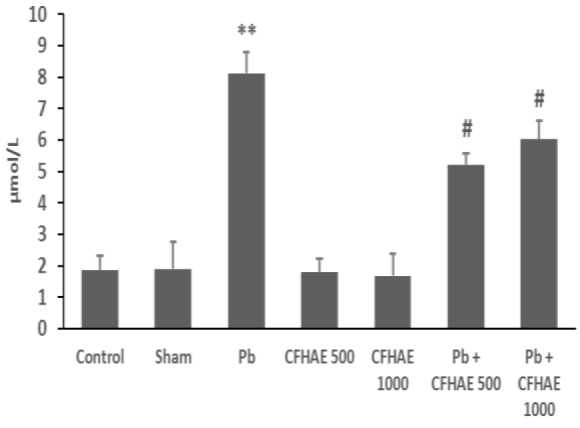
The serum mean levels MDA in different groups. Values represent means±SEM. **p<0.01 vs. control; # p<0.05 vs. Pb

The animals that were only treated with Pb had a significant (p<0.01) reduction in serum MDA level compared to the control group. However, co-administration of CFHAE and Pb, significantly (p<0.05) increased MDA levels (Figure 5).

**Effects on sex hormones**

Hormonal evaluations showed a significant (p<0.01 for all cases) decrease in concentrations of sex hormones in Pb group compared to control and sham groups while co-administration of CFHAE with Pb significantly increased sex hormones levels in comparison to Pb group (p<0.05 to p<0.01) (Table 5).

**Table 3:** Comparison of sperm parameters in different groups

**Pb+CFHAE 1000**	**Pb+CFHAE 500**	**CFHAE 1000**	**CFHAE 500**	**Pb**	**Sham**	**Control**	**Groups**
20.42±1.02^##^	19.17±1.44^##^	27.15±1.09	28.13±1.63	11.77±0.81^**^	26.35±1.20	27.11±1.36	**Sperm count (10** ^6^ **)**
75.94±1.49^##^	70.03±1.27^##^	83.71±1.50	82.28±0.77	57.84±1.16^**^	81.10±1.82	81.08±0.95	**Sperm viability (%)**
60.71±1.05^##^	63.50±1.38^##^	78.65±0.61	80.34±0.90^*^	51.49±0.78^**^	75.28±1.21	76.24±1.33	**Sperm motility (%)**
85.97±1.22^##^	86.34±0.68^##^	91.20±1.01	89.47±1.40	76.48±1.29^**^	88.23±0.58	90.37±1.71	**Sperm morphology (%)**
9.14±1.23^##^	13.59±0.96^#^	3.04±1.15	3.08±0.71	18.79±0.48^**^	3.24±0.41	3.08±0.61	**DNA damage (%)**
1.35±0.35^##^	1.60±0.41^#^	1.13±0.74	1.11±0.30	1.89±0.59^**^	1.14±0.37	1.12±0.32	**TZI (%)**

TZI: Teratozoospermia index. Values represent means±SEM. *p<0.05, **p<0.01 vs. control; #p<0.05, ##p<0.01 vs. Pb.

**Table 4:** The mean amounts of antioxidant activity and lipid peroxidation in different groups

**Groups**	**Control**	**Sham**	**Pb**	**CFHAE 500**	**CFHAE 1000**	**Pb+CFHAE 500**	**Pb+CFHAE 1000**
**TAC (μmol/L)**	3.21±0.81	3.18±0.70	0.41±0.29^**^	3.32±0.43	3.45±0.38^*^	0.97±0.41^##^	1.61±0.37^##^
**GSH (U/L)**	81.19±1.48	80.37±0.56	44.25±1.17^**^	81.08±1.79	81.75±1.22	54.69±0.44^##^	62.95±1.62^##^
**SOD (U/L)**	1637±1.39	1620±1.81	1124±1.72^*^^*^	1652±1.05	1681±1.19	1318±1.30^##^	1394±0.93^##^
**CAT (U/L)**	1.29±0.39	1.28±0.33	0.64±0.41^**^	1.30±0.61	1.37±0.56	0.85±0.42^#^	1.04±0.33^##^
**GPx (U/L)**	7.45±1.12	7.41±1.75	2.37±1.02^**^	7.42±1.34	7.63±1.60	4.85±0.37^##^	5.30±0.51^##^

TAC: Total antioxidant capacity, GSH: Glutathione SOD: Superoxide dismutase, CAT: Catalase, GPx: Glutathione peroxidase. Values represent means±SEM. *p<0.05, **p<0.01 vs. control; #p<0.05, ##p<0.01 vs. Pb.

**Table 5:** The serum mean concentrations of sexual hormones in different groups

**Group**s	**Control**	**Sham**	**Pb**	**CFHAE 500**	**CFHAE 1000**	**Pb+CFHAE 500**	**Pb+CFHAE 1000**
**FSH (mIU/ml)**	4.40±0.18	4.38±0.18	1.52±0.18^**^	4.49±0.18	4.45±0.18	2.95±0.18^##^	3.12±0.18^##^
**LH (mIU/ml)**	3.20±0.66	3.21±0.66	1.64±0.66^**^	3.29±0.66	3.41±0.66	2.35±0.66^#^	2.70±0.66^##^
**Testosterone (μmol/L)**	6.07±1.49	5.89±1.49	3.19±1.49^**^	6.11±1.49	6.28±1.49	4.79±1.49^##^	5.10±1.49^##^

Values represent means±SEM. **p<0.01 vs. control; #p<0.05, ##p<0.01 vs. Pb.

**Histopathological findings**

**Figure 6:**
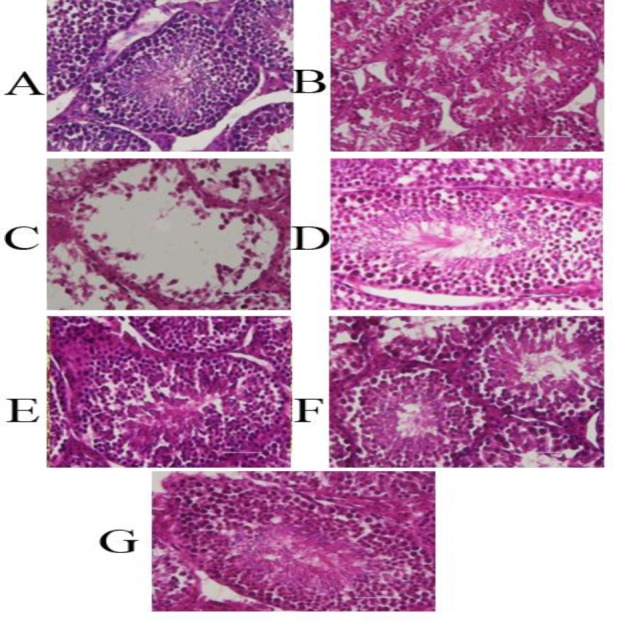
Light micrographs of testis tissue exhibiting protective effects of co-administration of CFHAE against Pb toxicity in testis. (A): Control, (B): Sham. (C): Pb, (D): CFHAE 500, (E): CFHAE 1000, (F): Pb+CFHAE 500, and (G): Pb+CFHAE 1000. H&E staining (×400)

In the control group, seminiferous tubules, interstitium, Leydig cells and stromal elements have normal structures. Seminiferous tubules lined by multilayered epithelium contained Sertoli cells, spermatogonia (type A and B), primary spermatocytes, secondary spermatocytes, spermatids and spermatozoa (Figure 6A). The sham group was similar to the control group and had a normal structure (Figure 6B). In the Pb group, seminiferous tubules were thin with the dilated lumen and specific degenerative changes. The basal membrane was irregular and disrupted. The Leydig cells were reduced in interstitial tissue. Spermatogenic cells which included spermatogonia, spermatocyte and spermatids, were decreased and remaining cells had pyknotic nuclei and no Sertoli cells were observed. Spermatozoa were not present in the lumen which indicated suppression of spermatogenesis. Exfoliated cells were obvious in the lumen (Figure 6C). In groups treated with CFHAE 500 and 1000 mg/kg, seminiferous tubules with a normal different type of germ cells were seen (Figures 6D and E). In the group treated with Pb and CFHAE 500 mg/kg/day, degenerative changes caused by Pb were reduced after treatment with CFHAE. Also, increase of spermatogonia cells in seminiferous tubules is obvious (Figure 6F). In the group treated with Pb and CFHAE 1000 mg/kg, germinal cells in all the tubules were increased. Basement membrane structure was normalized and marked increases in spermatozoa in the lumen can be seen (Figure 6G).

**Effects on expression of** ***Nrf2*** **and** ***iNOS*** **genes in testis**

**Figure 7:**
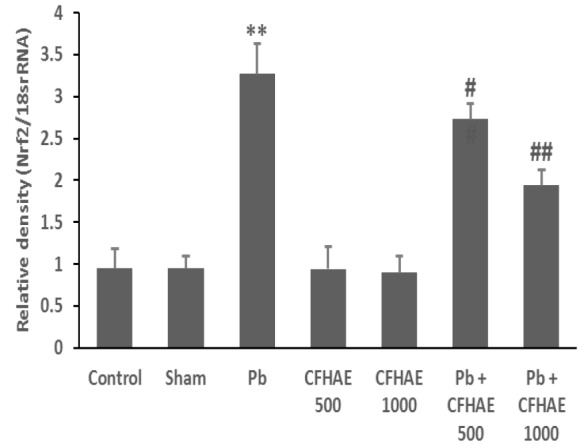
The densitometry analyses of Nrf2, which were normalized, based on the expression level of 18srRNA. **p<0.01 vs. control; #p<0.05, ##p<0.01 vs. Pb

The results in Figure 7 show that Pb significantly (p<0.01) increased *Nrf2* expression compared with control group. Also, co-administration of CFHAE with Pb significantly (p<0.05 at CHFAE 500 mg/kg and p<0.01 at CHFAE 1000 mg/kg) decreased its expression compared with Pb group. 

**Figure 8:**
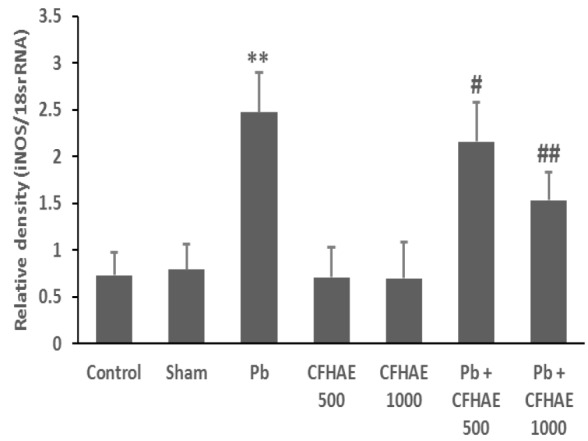
The densitometry analyses of iNOS, which were normalized, based on the expression level of 18srRNA. **p<0.01 vs. control; #p<0.05, ##p<0.01 vs. Pb

Data in Figure 8 show that Pb group exhibited a significant (p<0.01) increase in *iNOS* expression in comparison with the control and sham groups. However, co-administration of CFHAE with Pb significantly (p<0.05 at CHFAE 500 mg/kg and p<0.01 at CHFAE 1000 mg/kg) decreased the expression of *iNOS* compared to Pb group. 

## Discussion

In the present study, our findings indicated that administration of CFHAE improved sperm parameters, elevated sex hormones, TAC, GSH content and antioxidant enzymes activity of serum, decreased serum MDA levels, down-regulated testicular expression of *Nrf2* and *iNOS* genes, and also ameliorated histopathological alterations in testis tissue caused by Pb.

Similar to previous studies (Dorostghoal et al., 2014 ▶; Mabrouk and Ben Cheikh, 2014 ▶; Reshma Anjum et al., 2017 ▶, 2011), our results showed that Pb attenuates sperm parameters. These findings may be caused as Pb can passes through the blood-testis barrier (Creasy, 2001 ▶), induces oxidative stress and causes lipid peroxidation and DNA damages (Zhang et al., 2014) which results in impairment of spermatogenesis. Since testis tissue possesses high content of polyunsaturated lipids in its cells' membrane, it is susceptible to oxidative stress (Mishra and Acharya, 2004 ▶). Products of lipid peroxidation detrimentally influences membrane function and motility of sperms which leads to diminishing their fertility potential (Choudhary et al., 2010 ▶).

Administration of Pb decreased serum TAC, GSH content and activity of antioxidant enzymes. The mechanism through which Pb altered antioxidant enzymes activity, is mediated by direct inhibition of functional SH groups in antioxidant enzymes and/or by binding to metal cofactors of these enzymes such as Cu, Zn and Mn (Patra et al., 2011 ▶). Also, MDA level increased in Pb group. These results are in line with prior reports (Dkhil et al., 2016 ▶; Dorostghoal et al., 2014 ▶; Patra et al., 2011 ▶; Reshma Anjum et al., 2017 ▶).

Effects of Pb on LH and FSH are contradictory. As some studies, in line with the present research, reported a reduction of these hormones following exposure to Pb (Ayinde et al., 2012 ▶; Dorostghoal et al., 2014 ▶), other studies reported increases in them (Anjum and Reddy, 2015 ▶). The difference in various reports is attributed to the factors such as Pb concentration, exposure interval, and physiological status of the testis and reproductive axis (Gandhi et al., 2017 ▶).

The reduction in the concentration of serum testosterone in the present research, is in accordance with prior studies (Anjum and Reddy, 2015 ▶; Dkhil et al., 2016 ▶; Mabrouk and Ben Cheikh, 2014 ▶; Reshma Anjum et al., 2017 ▶). Possibly, Pb exposure adversely affected hypothalamic–pituitary–gonadal axis (Gandhi et al., 2017 ▶). Researchers revealed that exposure to Pb degenerates gonadotrophic cells of the pituitary gland (Hamadouche et al., 2013 ▶). Moreover, Pb induces extrinsic apoptosis signal pathway in Leydig cells (He et al., 2017 ▶). Oxidative damages make Leydig cells less sensitive to LH which can affect testosterone synthesis (Glade and Smith, 2015 ▶). Pb suppresses the activity of steroidogenic enzymes in Leydig cells. Thereby, another reason of decrement of testosterone might be due to inhibition of androgen synthesis by Pb (Huang and Liu, 2004 ▶; Liu et al., 2001 ▶). 

Exposure to Pb induced expression of *iNOS* gene in testis tissue. iNOS catalyzed the synthesis of nitric oxide (NO) from L-arginine (Aktan, 2004 ▶). NO incorporates with superoxide anion in the formation of peroxynitrite anion (ONOO^−^), a strong ROS which is able to cause membrane lipid peroxidation (Moneim, 2015 ▶). Dkhil et al. (2016) ▶ reported that Pb exposure increases generation of NO in rats' which is in line with our results.

Nrf2 is the primary regulator of antioxidant response and participates in protection against oxidative stress (Lindl and Jordan-Sciutto, 2008 ▶). Nrf2 induces the expression of phase II antioxidant enzymes in the nucleus (Jung and Kwak, 2010 ▶). It has been found that defective Nrf2 signaling causes spermatogenesis defects (Yu et al., 2012 ▶). Exposure to heavy metals results in activation of the Nrf2 pathway (Simmons et al., 2011 ▶). Our study indicated that Pb up regulates *Nrf2* gene expression. Wang et al. (2012) ▶ showed that exposure to Pb up-regulates expression of Nrf2 in testis which is in agreement with our results.

To the best of the authors knowledge, the present research is the first study on ameliorative effects of CFHAE against Pb-induced reproductive toxicity. The present research showed that co-administration of CFHAE improved sperm parameters in animals treated with Pb. Similar to our findings, prior studies showed that carob aqueous extract has beneficial effects on sperm parameters in experimental animals (Ata et al., 2018 ▶; Vafaei et al., 2018 ▶). These effects may be due to antioxidant properties of carob fruit. CFHAE enhanced antioxidant activity of serum which is similar to data reported by Rtibi et al. (Pedersen, 2001 ▶). Also, many studies indicated that administration of carob increases antioxidant enzymes in different organs (Abdel-Rahman et al., 2018 ▶; Rtibi et al., 2016a ▶, 2016b ▶, 2015; Suzek et al., 2017 ▶). 

The antioxidant ability of CFHAE might be related to its high phenolic and flavonoid contents. Phytochemical screening in this study showed that CFHAE possesses gallic acid, caffeic acid, chlorogenic acid, rutin, coumaric acid quercetin, cinnamic acid and apigenin which have antioxidant activity.

Besides, carob fruit contains trace elements such as Fe, Cu, Mn and Zn (Özcan et al., 2007 ▶; Oziyci et al., 2014 ▶) which are co-factors of antioxidant enzymes (Harris, 1992 ▶) which boost antioxidant system in Pb exposure. The MDA levels in serum decreased in Pb + CFHAE groups compared to Pb group which indicates that administration of CFHAE reduces lipid peroxidation. It could be a consequence of the reduction of cell damages due to neutralization of free radicals by CFHAE's antioxidant compounds. Similar to this research, several studies showed that administration of carob aqueous extract decreased MDA levels (Rtibi et al., 2016a ▶; Vafaei et al., 2018 ▶).

Data from real time PCR revealed that *Nrf2* expression was down-regulated in Pb + CFHAE groups which demonstrated that co-administration of CFHAE reduced oxidative stress in animals exposed to Pb. This is the first study that demonstrated CFHAE decreased expression of *Nrf2*. Also, co-administration of CFHAE with Pb down-regulated *iNOS* which is not reported so far and indicates that CFHAE decreases the production of NO. In line with the present results, Al-Olayan et al. (2016) ▶ showed that carob pod aqueous extract reduced NO level in the liver of mice experimentally infected with *Schistosoma mansoni*. Abdel-Rahman et al. (2018) ▶ showed that treatment of rats exposed to water pipe smoke, with carob aqueous extract decreased NO levels in lung tissue.

Other researchers reported protective potential of carob pods against oxidative stress which is similar to our findings (Abdel-Rahman et al., 2018 ▶; Rtibi et al., 2016a ▶, 2016b ▶, 2015; Suzek et al., 2017 ▶; Vafaei et al., 2018 ▶).

Co-administration of CFHAE elevated sex hormones in groups that were simultaneously exposed to Pb. Increases in LH and FSH levels might be due to protecting effects of CFHAE on endocrine cells of the pituitary gland since Pb attenuates antioxidant enzymes and causes degenerative changes in these cells (Hamadouche et al., 2013 ▶). In line with our results, previous researches elucidated that administration of carob extract increases serum concentrations of LH and testosterone (Mokhtari et al., 2012 ▶; Vafaei et al., 2018 ▶). Oxidative stress reduces antioxidant activity and increases lipid peroxidation in Leydig cells which results in impairment of testosterone synthesis (Glade and Smith, 2015 ▶). Furthermore, increases in testosterone levels in Pb + CFHAE groups occurred because CFHAE decreases oxidative stress caused by Pb in Leydig cells.

This study demonstrated that CFHAE alleviates Pb toxicity in the reproductive system by diminishing the development of oxidative stress. Hence, it can be utilized as an antioxidant agent in the diet of infertile male patients and the people who may have chances of occupational and environmental exposure to heavy metals such as Pb and other agents that induce oxidative stress. 
